# A Quasi-Experimental Evaluation of a Primary Care Behavioral Health Integration Program Based on the Chronic Care Model

**DOI:** 10.1007/s11606-025-09641-0

**Published:** 2025-06-06

**Authors:** Neda Laiteerapong, Sandra A. Ham, Mim Ari, Nancy Beckman, Lisa M. Vinci, Fabiana S. Araújo, Daniel Yohanna, Danica Moser, Vivek Nandur, Erin M. Staab

**Affiliations:** 1 Department of Medicine, University of Chicago, Chicago, IL USA; 2 Department of Psychiatry and Behavioral Neuroscience, University of Chicago, Chicago, IL USA; 3 Sandra Ham Consulting, Buffalo, NY USA; 4 Booth School of Business, University of Chicago, Chicago, IL USA

**Keywords:** Behavioral health, Primary care, Clinical decision support, Integrated care, Chronic care model

## Abstract

**Background:**

Mental health conditions are often underdiagnosed and undertreated in primary care, particularly in underserved areas. Integrated behavioral health models can address this gap, but their reliance on mental health professionals may limit scalability. A multi-level intervention based on the chronic care model may enhance mental health care delivery in resource-limited settings.

**Objective:**

To evaluate the effectiveness of a chronic care model–based primary care behavioral health integration program for improving the diagnosis and management of mental health conditions in a primary care setting.

**Design:**

Quasi-experimental, pre-post observational study using interrupted time series analysis over a 10-year period (2010–2019).

**Participants:**

In total, 59,723 adult patients aged >18 who had at least two medical visits between 2010 and 2019. The patient population was 58% non-Hispanic Black, 29% non-Hispanic White, and 64% female.

**Interventions:**

Implementation of clinical decision support systems for common mental health conditions (e.g., depression, anxiety, ADHD), self-management support, delivery system re-design within integrated behavioral health services, and health system community support with weekly behavioral health tips.

**Main Measures:**

Changes in the rate of mental health diagnoses and follow-up care (including psychiatric medications, referrals to psychiatry or behavioral medicine, and primary care visits with a mental health diagnosis).

**Key Results:**

The rate of mental health diagnoses increased by 58.8 per 1000 person-years in the first year after intervention implementation (*p* = 0.001). Follow-up care in primary care increased by 102.1 per 1000 person-years (*p* = 0.03), while psychiatry referrals decreased by 59.8 per 1000 person-years annually after the intervention (*p* = 0.004).

**Conclusions:**

This chronic care model-based system-level intervention was associated with significant increases in mental health diagnosis and treatment within primary care. Expanding the role of primary care in managing mental health conditions may offer a scalable solution to mental health professional shortages, especially in underserved areas.

**Supplementary Information:**

The online version contains supplementary material available at 10.1007/s11606-025-09641-0.

## INTRODUCTION

Since the 1970 s, primary care (PC) has been described as the *de facto* home for the diagnosis and treatment of most mental health problems.^1^ In the USA, primary care providers (PCPs) manage most patients with mental health conditions.^2,3^ However, PCPs fail to diagnose many mental health conditions, and even after diagnosis, PCPs may not prescribe sufficient dosages of psychiatric medications for disease remission.^4,5^ Integrating behavioral health (BH) professionals into PC clinics is an effective strategy to increase diagnosis and treatment of mental health problems.^6–11^

Two prevailing models of integrated BH care exist: the primary care behavioral health (PCBH) and collaborative care model (CoCM).^7,12^ These models are similar in their emphasis on the addition of BH professionals (BHP), team-based care, and collaborative management. The PCBH model employs a BH professional to provide brief evidence-based treatments within the PC setting.^12^ The PCBH model allows for integrated care for a broad set of BH problems and has been shown to improve clinical outcomes.^12–21^ The CoCM relies on BH care managers and a consultative psychiatrist and emphasizes measurement-based care (the use of brief symptom screening questionnaires to guide decisions about treatment and triage) and use of a population health registry.^22^ CoCM has been shown to improve mental health symptoms and lead to treatment remission in multiple clinical settings.^10^

While these care models are effective, one shortfall is their reliance on the addition of BHPs to the PC team. The volume of BH problems in PC is high, such that having a sufficient number of BHPs trained in integrated BH may be infeasible.^23^ Additionally, it can be challenging to hire BHPs^24^ since most people in the USA live in Mental Health Professional Shortage Areas.^25^ Furthermore, PCBH and CoCM implementations are hampered by PCP resistance to change and lack of awareness of their advantages.^26,27^

Because of limited BHP access, a realistic strategy for improving BH management in PC must include expanding the role of PCPs; however, PCPs are already very busy.^28^ We sought to design and implement a multi-component system-level intervention based on the Chronic Care Model to facilitate BH care in PC. We describe the 5-year pre-post impact of our intervention on BH diagnoses and treatment at a large academic PC clinic.

## METHODS

### Setting

The setting was the academic general internal medicine practice at UChicago Medicine. The clinic is staffed by 36 attending physicians, 4 advanced practice nurses, 100 internal medicine residents, and 16 internal medicine-pediatric residents, for a total of ~18 full-time equivalents (FTEs). The population was ~30,000 patients, with a race/ethnicity breakdown of 58% Black, 28% White, 5% Asian, 5% Hispanic, 4% Other/Unknown and an insurance mix of 47% private insurance, 45% Medicare, and 8% Medicaid. The clinic is in the South Side of Chicago and surrounded by both PC and Mental Health Professional Shortage Areas. The catchment area has ~15 psychiatrists/100,000 residents, and 80–99% of people with psychological illness do not see a mental health professional.^29^

### Intervention

In 2015, an interdisciplinary team launched the Primary Care-Behavioral Health Integration Program (PC-BHIP) at UChicago Medicine to integrate BH into our academic internal medicine teaching practice.^30^ PC-BHIP had a broad 5-year goal of improving BH outcomes by increasing the provision of BH care in the PC clinic. We used continuous quality improvement to advance BH integration.^30,31^ Interventions were anchored on the Chronic Care Model,^32^ which emphasizes redesign of practice systems and roles, clinical decision support, clinical information systems, and patient self-management support. The chronic care model is an effective model for improving the quality of care in PC, which has been used in the context of BH conditions.^33^ In particular, CoCM is based on the chronic care model. However, our health system did not have the personnel to implement CoCM in 2015, and we were interested in broader PC system change beyond specific BH conditions and populations for which CoCM had been proven effective.

**Figure 1:**
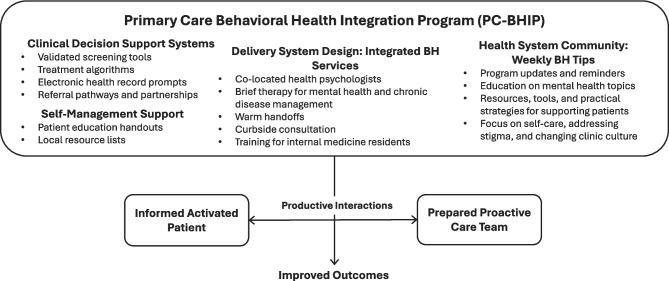
Conceptual model of primary care–behavioral health integration program.

Figure 1 describes our intervention. We developed new clinical decision support systems for a wide range of BH conditions, patient self-management education handouts and resource lists, an integrated BH service, and enhanced referral pathways and partnerships.

Each clinical decision support system emphasized the measurement-based care approach. Measurement-based care relies on the use of screening tools that can provide a reliable measure of symptoms to triage patients to the appropriate intensity of treatment.^34^ This approach has been demonstrated to improve symptoms more than usual care.^35^ Thus, our clinical decision support systems included access to validated brief screening tools and one-page comprehensive summaries for how to screen, manage, and follow-up; initiate and titrate medications; use adjunctive treatments; and when to refer to higher intensity care. We released tools for major depressive disorder in 2016 and integrated depression screening into the electronic health record (EHR) and medical assistant workflow. We have previously published on our efforts to increase depression screening rates over time.^36–38^ After depression, driven by clinical need, we developed systems for panic (2016), anxiety (2018), attention deficit hyperactivity disorder (ADHD) (2018), and post-traumatic stress disorder (PTSD) (2018). Current versions of our clinical decision support tools are available in the Supplement (Supplemental Figure 1 A-1E). As clinical decision support systems were rolled out, we developed patient self-management education and resource list handouts.

To address system redesign, we established a PCBH clinic with a clinical health psychologist who trained psychology interns, externs, and post-doctoral fellows in the academic general internal medicine practice in 2015. The PCBH team offered brief therapy in English and Spanish, warm handoffs, curbside consultation, classroom lectures on mental health, and one-on-one training for internal medicine PGY1 s. The PCBH clinic was small, totaling 0.25 to 0.30 FTEs during the study period.

For patients in need of higher intensity treatments, we improved our referral network. First, we strengthened internal referral pathways with the Department of Psychiatry and Behavioral Neuroscience. Then in 2018, we established relationships with community BH organizations for closed loop referrals, meaning they agreed to receive referrals from our institution, contact our patients, and inform us if patients were scheduled for appointments. Intrinsic to these partnerships was a mutual respect for existing workflows and a low burden to entry. At the end of 2019, we had partnerships with seven community BH organizations.

Lastly, in January 2016, we began to disseminate weekly tip emails to PCPs to remind them about our resources and share knowledge about BH conditions, well-being, health behavior change, BH care, treatments, and community mental health resources. These weekly emails also featured evidence-based recommendations for self-care and discussed mental health stigma.

### Study Design

We conducted a quasi-experimental evaluation of our program, using an interrupted time series analysis that compared retrospective data from 5 years prior to our quality improvement intervention to the first 5 years after our intervention. Observational data were provided by the Clinical Research Data Warehouse (CRDW) maintained by the Center for Research Informatics (CRI) at the University of Chicago. The study was approved by the University of Chicago Biological Sciences Division Institutional Review Board.

### Participants

We included all adult patients aged 18 years or older seen in the clinic with at least two medical visits between 2010 and 2019. We required at least two visits to reduce noise created by patients who were seen in the clinic for only a single visit and were not receiving PC in the clinic.

### Outcomes

Our outcomes focused on effectiveness, defined by changes in mental health diagnosis and management. For diagnosis, we examined the change in the rate of patients seen in the PC clinic who received a mental health visit diagnosis. We used International Classification of Diagnosis codes (Supplemental Table 1) to define mental health diagnoses overall and specifically for the conditions for which we had developed clinical decision support systems (depression, panic, anxiety, ADHD, and PTSD). We expected a larger effect on depression and panic compared to anxiety, ADHD, and PTSD because interventions for these latter conditions were not implemented until 2018.

For management, we examined the change in the rate of patients with a mental health visit diagnosis who received mental health treatment. Treatment outcomes included rates of new psychiatric medication prescription, referral to a mental health professional (psychiatry department or PCBH), visit to a mental health professional (psychiatry or PCBH), PCP visit with documented mental health visit diagnosis, as well as combinations of psychiatry, PCBH, and/or PCP visits for mental health diagnoses. A new psychiatric medication prescription was defined by an initial prescription after a 1-year look-back.

### Statistical Analysis

Summary statistics were calculated to describe the sample groups. An interrupted time series analysis was used to compare each of the mental health visit diagnoses and management treatments for the 5 years pre-intervention (2010–2014) vs. post-intervention (2015–2019). The unit of analysis was the visit. For each outcome, we show the time series charts of summary data and model-based estimates. We report model-based estimates of mean rates of change (per 1000 person-years) for the pre-intervention interval, post-intervention interval, and year of change. Analyses were also stratified by birth cohort, sex, and race/ethnicity to explore disparate effects on diagnosis and management.

## RESULTS

During the 10-year period, there were 59,723 adult patients (pre-BHIP: 28,981 (49%) and post-BHIP: 30,742 (51%)) (Table 1) who had a total of 409,639 PC visits (pre-BHIP: 205,906 and post-BHIP: 203,733). Analyses included a total of 179,247 person-years (pre-BHIP: 89,034 and post-BHIP: 90,214 person-years); patients were counted once per year that they had a visit, for up to 10 person-years each. About one-third of patients were born before 1950 (32%), 45% were born between 1950 and 1974, and 23% were born in 1975 or later. Over 60% were female. The majority were non-Hispanic Black/African American (58%); non-Hispanic white adults were 29% of the population. The mean number of PC visits per patient was 6.8 (standard deviation (SD), 5.8); during the pre-intervention, patients had slightly more visits (mean, 7.1; SD, 5.9) than post-BHIP (6.6, SD 5.6).

**Table 1:** Study Population Demographics Before and After Primary Care–Behavioral Health Integration Program (PC-BHIP) (2009–2019)

	Before PC-BHIP		After PC-BHIP		Overall^a^	
	*N* or mean	% or SD	*N* or mean	% or SD	*N* or mean	% or SD
Total patients, *N* (%)	28,981	100	30,742	100	59,723	100
Person-years	89,034	100	90,214	100	179,247	100
Year of birth, *N* (%)						
≥1975	5951	21	7725	25	13.676	23
1950–1974	12,891	44	14,236	46	27,127	45
<1950	10,139	35	8781	29	18,920	32
Sex, *N* (%)						
Female	18,637	64	19,369	63	38,006	64
Male	10,344	36	11,373	37	21,717	36
Race/ethnicity, *N* (%)						
Non-Hispanic Black/African American	16,772	58	18,100	59	34,872	58
Non-Hispanic White	8370	29	8733	28	17,103	29
Hispanic or Latino	1131	4	1435	5	2566	4
Non-Hispanic Asian/Mideast Indian	1490	5	1612	5	3102	5
Non-Hispanic other and unknown	1218	4	862	3	280	3
Total visits, *N* (%)	205,906	100	203,733	100	409,639	100
Visits per patient, mean (SD)	7.08	5.93	6.61	5.63	6.84	5.78

*SD*, standard deviation

^a^The overall N is less than the sum of the pre-BHIP and post-BHIP groups because some patients had clinic visits during both the pre- and post-BHIP periods

**Figure 2:**
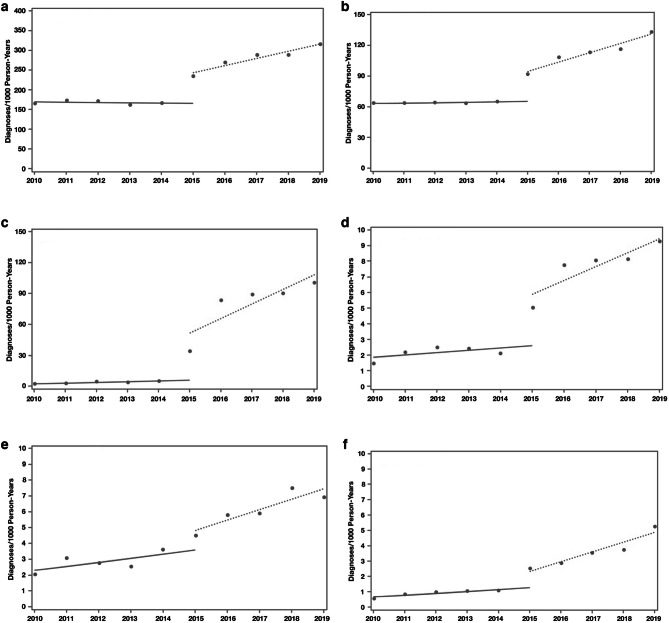
Changes in mental health visit diagnoses in primary care before and after primary care–behavioral health integration program. **a** Any mental health condition. **b** Depression. **c** Anxiety disorders. **d** Panic disorder. **e** Attention deficit hyperactivity disorder. **f** Post-traumatic stress disorder. Circle: Before primary care–behavioral health integration program. Solid line: Trend before primary care–behavioral health integration program. Square: After primary care-behavioral health integration program. Dashed line: Trend after primary care–behavioral health integration program.

The rate of visits with a mental health diagnosis increased post-BHIP. The interrupted time series analysis demonstrated that the implementation of BHIP changed visit diagnoses in the first year after implementation and increasingly over time (Table 2, Fig. 2). The rate of mental health diagnoses during the pre-BHIP period was 169.5 per 1000 person-years. The rate of any mental health diagnoses was stable (−0.7 (−5.6 to 4.3) per 1000 person-years, *p*=0.80), as were rates of each of the examined mental health conditions. In the first year of BHIP, the rate of any mental visit diagnosis increased by 58.8 (38.4–79.1) per 1000 person-years (*p*=0.001). Rates were significantly higher for depression (20.7 (12.1–29.3) per 1000 person-years, *p*=0.003) and panic disorder (2.6 (0.9–4.2) per 1000 person-years, *p*=0.02), but non-significantly higher for anxiety. Rates of PTSD and ADHD did not change initially. During the first 5 years of BHIP, the annual rate of mental health diagnoses increased by 18.3 (13.3–23.2) per 1000 person-years (*p*<0.001) per year, as did the rates of each of the individual mental health conditions. The trend for continued annual increases in rates of mental health diagnoses was the greatest for anxiety disorders (14.0 (6.9–11.2) per 1000 person-years, *p*=0.008) per year.

**Table 2:** Trends in Mental Health Visit Diagnosis at Primary Care Visits Before and After Primary Care–Behavioral Health Integration Program (PC-BHIP)

	Mean rate before PC-BHIP (2010–2014)	Mean change per year before PC-BHIP (2010–2014)		Mean change in year of PC-BHIP implementation (2015)		Mean change per year after PC-BHIP (2016-2019)	
	Per 1000 person-years	Per 1000 person-years	*p*-value	Per 1000 person-years	*p*-value	Per 1000 person-years	*p*-value
Any mental health diagnosis	169.5 (152.3–186.8)	–0.7 (–5.6 to 4.3)	0.80	58.8 (38.4–79.1)	0.001	18.3 (13.3–23.2)	<0.001
Depression	62.9 (55.6–70.2)	0.4 (–1.7 to 2.5)	0.72	20.7 (12.1–29.3)	0.003	9.1 (7.0–11.2)	<0.001
Anxiety	1.7 (0–26.5)	0.7 (–6.4 to 7.8)	0.86	32.5 (3.4–61.7)	0.07	14.0 (6.9–21.1)	0.008
Panic disorder	1.7 (0.3–3.1)	0.2 (–0.2 to 0.6)	0.48	2.6 (0.9–4.2)	0.02	0.9 (0.5–1.3)	0.005
PTSD	0.6 (0–1.2)	0.1 (–0.1 to 0.3)	0.21	0.6 (–0.2 to 1.3)	0.18	0.6 (0.5–0.8)	<0.001
ADHD	2.0 (0.9–3.2)	0.3 (–0.1 to 0.6)	0.18	0.8 (–0.5 to 2.2)	0.28	0.7 (0.3–1.0)	0.008

Overall patterns in visit diagnoses were similar by sex, but changes in specific mental health diagnoses differed by sex post-BHIP. Females had higher rates of depression, anxiety, and panic disorders, and males had higher rates of ADHD diagnoses; PTSD rates were similar (Supplemental Figure 2). Similarly, overall trends in any mental health visit diagnoses were similar by birth cohort, but the youngest birth cohort had higher rates of diagnoses over time for depression, anxiety, panic, and ADHD (Supplemental Figure 3). In racial/ethnic subpopulation analyses, post-BHIP, the Hispanic subpopulation had a greater rate of any mental health visit diagnoses, which was reflected in each of the specific mental health conditions (Supplemental Figure 4).

**Figure 3:**
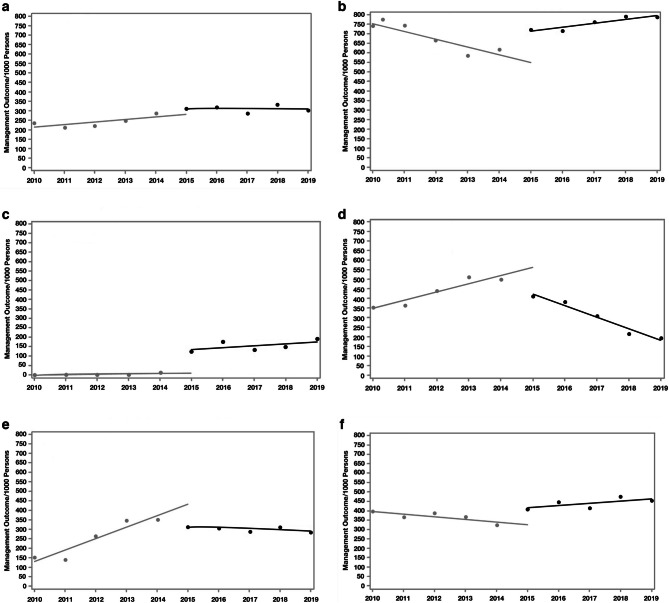
Changes in mental health treatment before and after primary care–behavioral health integration program. **a** Any follow-up encounter. **b** Primary care encounter. **c** Primary care behavioral health encounter. **d** Psychiatry encounter. **e** Initiation of psychiatric medications. **f** Any follow-up after psychiatric medications. Gray line: Trend before primary care–behavioral health integration program. Black line: Trend after primary care–behavioral health integration program.

After BHIP, BH services were more integrated into PC and PCBH (Fig. 3, Supplemental Table 2). Prior to BHIP, annual rates of follow-up in PC after mental health diagnoses were decreasing (−40.6 (−57.6 to −23.56) per 1000 person-years, *p*=0.003) at a similar rate as psychiatry visits were increasing (43.1 (27.5–58.7) per 1000 person-years, *p*=0.002). In the year after PC-BHIP implementation, the rate of follow-up increased in PC and PCBH visits (102.1 (32.0–172.2) per 1000 person-years, *p*=0.03, and 117.9 (69.8–166.0) per 1000 person-years, *p*=0.003, respectively). Over time, the rate of psychiatry visits decreased (−59.8 (−80.3 to −31.4) per year, *p*=0.004). Rates of psychiatric prescriptions had been increasing prior to PC-BHIP (52.0 (34.7–69.3), *p*=0.001), but after implementation, the rate of psychiatric medication prescriptions (−3.8 (−21.1 to 13.5), *p*=0.68) leveled off and no longer increased.

## DISCUSSION

In this observational, quasi-experimental study, implementation of a chronic care model-based integrated BH program in a large academic practice increased diagnoses and primary care treatment of BH conditions. Prior to our intervention, rates of mental health diagnoses were stable and psychiatric prescriptions and psychiatry visits were increasing. After our intervention, rates of mental health diagnoses increased, as did follow-up PC and BH visits, alongside a decrease in psychiatry visits and stabilization of psychiatric prescriptions.

A cornerstone of our intervention was the use of clinical decision support systems. Clinical decision support systems are an established strategy for enabling best practices with many different chronic conditions.^39^ However, only a few studies have examined the use of clinical decision support tools in PC, and those have yielded mixed results.^40–44^ In contrast to previous studies, our intervention focused on using measurement-based care and was developed by a multidisciplinary team (PC, psychology, psychiatry, health care administration, health services research) with expertise in health care worker burnout, clinical informatics, quality improvement, and implementation science. This accumulated expertise led to clinical decision support systems that provided highly practical information that emphasized ease of practice. We developed a wide range of tools and supports for each clinical decision support system, including easy access to brief screeners, access to internal and community BHPs, and weekly reminders about a wide range of BH topics and resources. These tools and resources are freely available on our website: https://voices.uchicago.edu/behavioralhealthintegrationprogram/.

Our intervention demonstrated a change in how PCPs treated mental health conditions. The majority of the change was through an increase in PC follow-up after mental health diagnoses, as opposed to an increase in visits to mental health professionals. This change in clinical practice is notable since prior literature has demonstrated that <5% of patients prescribed antidepressants saw their PCP over a 40-week follow-up period,^45^ and a lack of follow-up care is associated with lower adherence to antidepressant treatments.^46^ Thus, broadening the scope of PC practice to include BH conditions may improve patient outcomes. Our intervention also was associated with a decrease in psychiatric medications, which may have been due to earlier identification of less severe disease and/or increased use of therapy, as recommended by the clinical decision support tools. Importantly, integrated BH may have positive effects on PCPs as well, including less emotional exhaustion and burnout.^47^ Surveys of PCPs in our practice have demonstrated that they value this integrated BH model and its benefits.^30,31^ Similar benefits were described in a qualitative study of multiple stakeholders during the implementation of CoCM.^48^ In previous qualitative work, we have also demonstrated that patients valued our model of integration.^21^ In totality, our findings suggest that our program may have increased clinicians’ comfort with and provision of mental health care.

Because of the risk for interventions to exacerbate, rather than reduce, health care disparities, we analyzed our outcomes by several demographic characteristics. Results demonstrate the robustness of our intervention. For example, our analyses of changes in visit diagnoses by sex demonstrated overall no difference, though there were small differences that mirror known variation in disease prevalence (e.g., males having higher rates of ADHD).^49^ Similarly, the youngest birth cohort had higher rates of mental health diagnoses, similar to national trends,^50^ after our intervention. Because racial and ethnic minorities are more likely to receive mental health care in PC,^51^ our intervention serves as a potential model for reducing racial disparities in BH care.

Given our multi-level approach to BH integration— from clinical decision support from screening to management, self-management education and resources, integrated BH service, and health system community resources and support—it is difficult to determine which aspect was vital to its success. Further research would be useful to disentangle core versus optional components. Future studies should also examine changes in clinical symptoms. We did not explore changes in mental health symptom frequency, for example, because at the time of the study, documented re-measurement of the PHQ-9 occurred infrequently. Additionally, we could not assess whether there was a shift in the severity of mental health conditions managed by primary care or other health outcomes (e.g., hospitalizations, emergency department visits), which would be important to examine in the future.

This intervention was conducted at a single urban academic clinic, which affects its external validity. This setting has unique challenges, which may have impacted the effectiveness of this intervention. For example, some academic physicians (resident and faculty) have low clinical effort because of other responsibilities, which can lead to greater challenges assimilating changes in clinical practice. Resident physician turnover in an academic practice also adds complexity. Additionally, this model’s utility may be most helpful in settings where patients have difficulty accessing psychiatrists and therapists. In clinical settings where there are enough BHPs, PCPs may not feel compelled to expand their scope of practice or integrate BH. Lastly, this study was observational and examined the program through 2019 before the COVID-19 pandemic led to seismic shifts in health care, incidence of mental health conditions, mental health stigma, and acceptance of mental health treatment. While we used a quasi-experimental design to analyze the effects of our intervention, results should be interpreted with caution, and more recent analyses will be important to understand the long-term implications of this intervention.

This system-level chronic care model approach to integration of PC and BH was associated with a sizable increase in mental health diagnoses and treatment in PC. In contrast to other models of integrated BH, this model may be sustainable by changing the scope of practice in PC. Further research is needed to determine if implementation in other settings leads to similar outcomes.

## Supplementary Information

Below is the link to the electronic supplementary material.

Supplementary file1 (PPTX 1345 KB)

Supplementary file2 (DOCX 2343 KB)

## Data Availability

The datasets analyzed during the current study are not publicly available because they were gathered as part of usual clinical care and patient consent for data sharing was not provided.

## Declarations:

### Data Access, Responsibility, and Analysis:

Neda Laiteerapong had full access to all the data in the study and takes responsibility for the integrity of the data and the accuracy of the data analysis.
