# Supplementary material for: A Quasi-Experimental Evaluation of a Primary Care Behavioral Health Integration Program Based on the Chronic Care Model
Source: J Gen Intern Med. 2025 Jun 6;41(4):913–21. doi: 10.1007/s11606-025-09641-0 (PMC13009416; doi:10.1007/s11606-025-09641-0)
Supplement: Supplementary file 1 — Supplementary file1 (PPTX 1345 KB) [file 11606_2025_9641_MOESM1_ESM.pptx]

## Slide 1
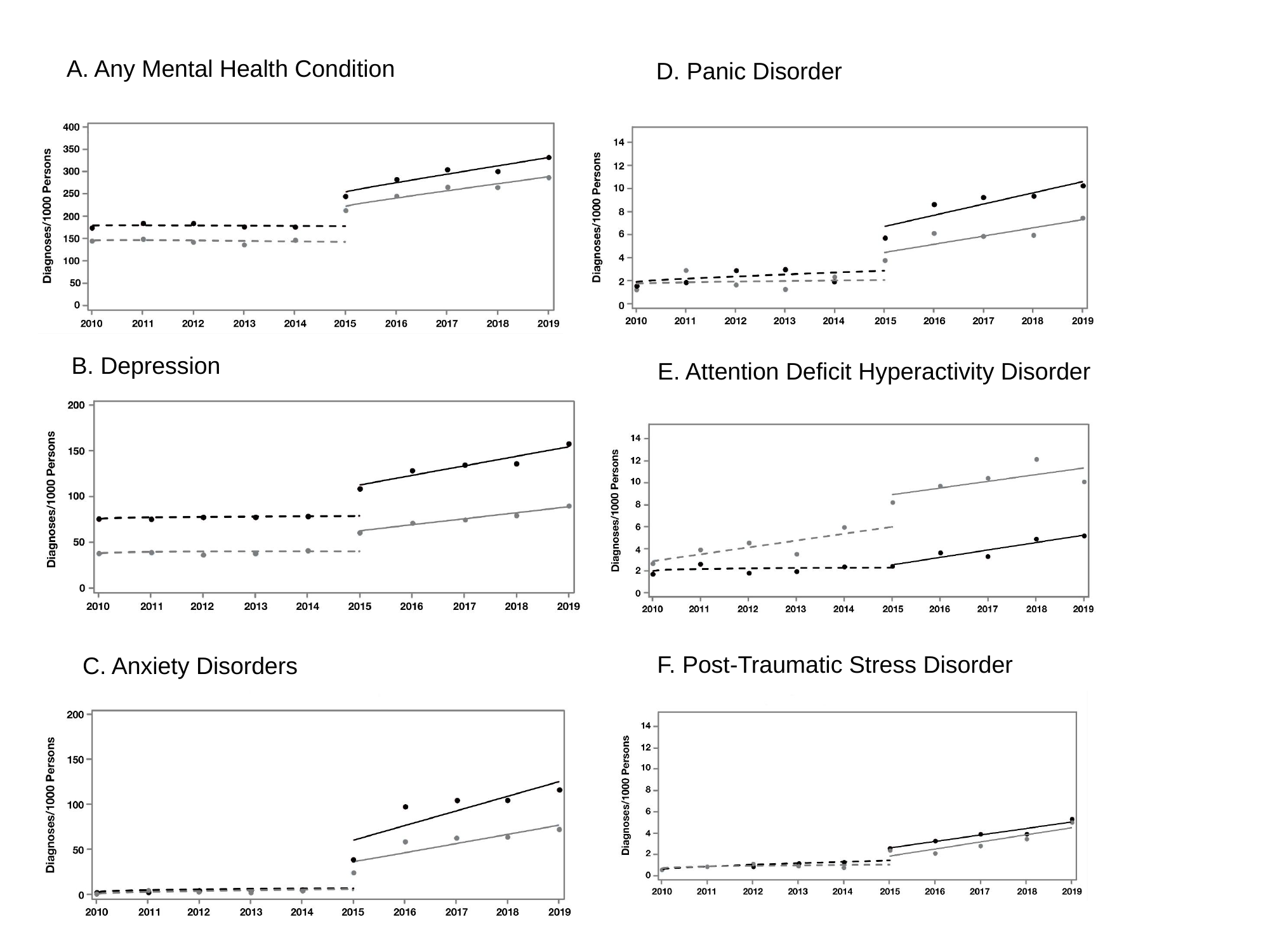

A. Any Mental Health Condition
D. Panic Disorder
B. Depression
E. Attention Deficit Hyperactivity Disorder
F. Post-Traumatic Stress Disorder
C. Anxiety Disorders

## Slide 2
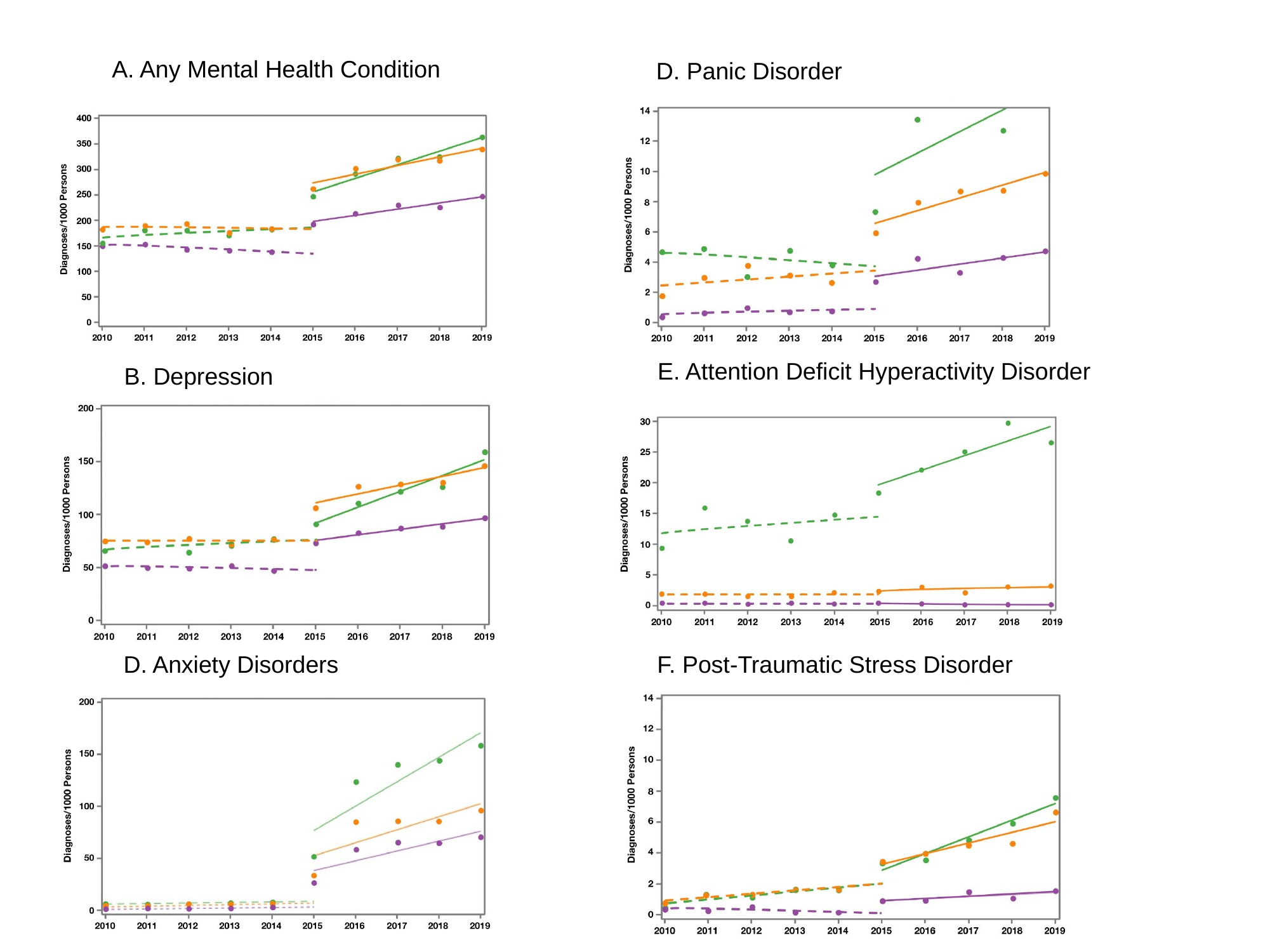

A. Any Mental Health Condition
D. Panic Disorder
E. Attention Deficit Hyperactivity Disorder
B. Depression
D. Anxiety Disorders
F. Post-Traumatic Stress Disorder

## Slide 3
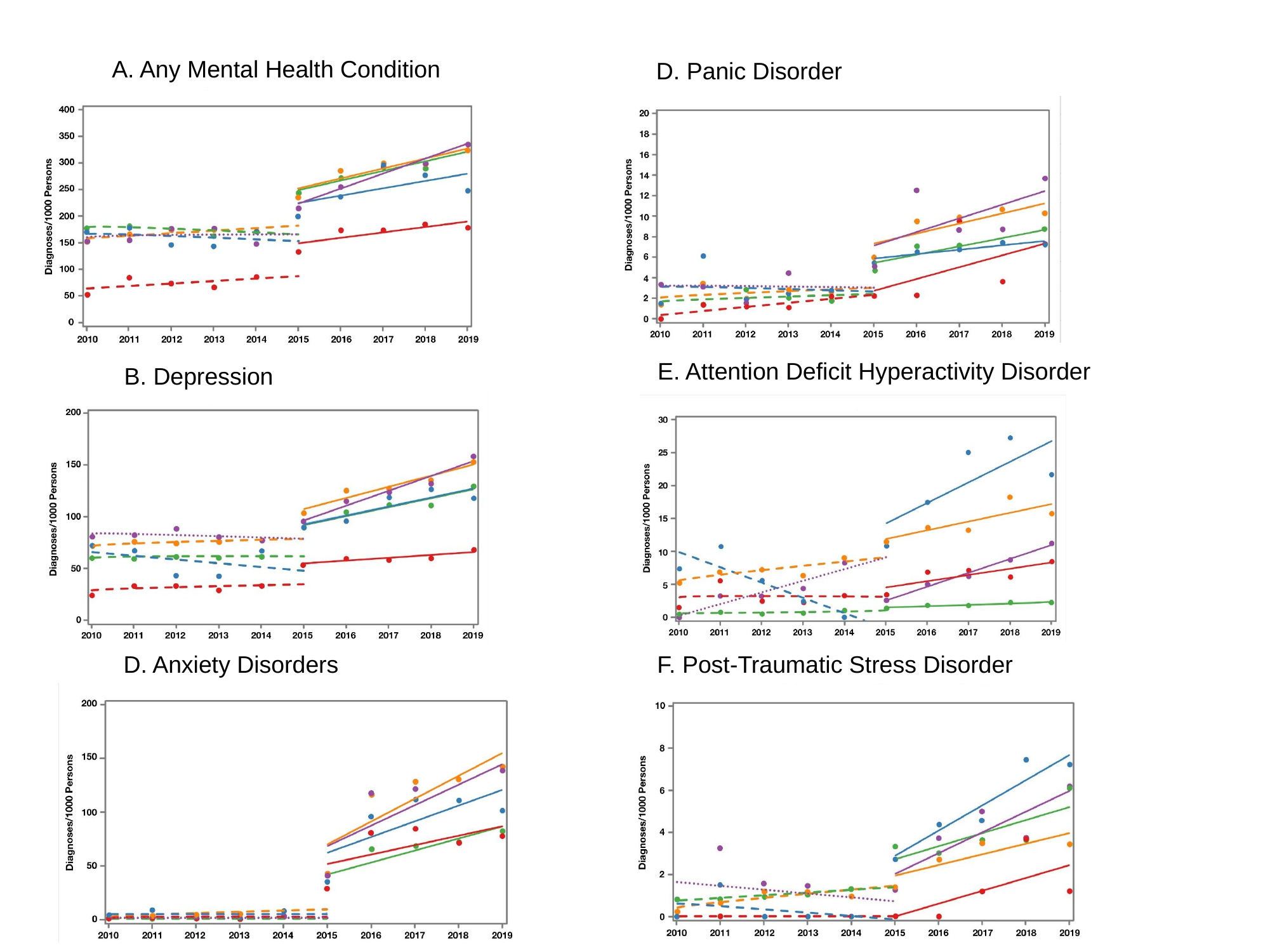

A. Any Mental Health Condition
D. Panic Disorder
E. Attention Deficit Hyperactivity Disorder
B. Depression
D. Anxiety Disorders
F. Post-Traumatic Stress Disorder
