# Supplementary material for: A Quasi-Experimental Evaluation of a Primary Care Behavioral Health Integration Program Based on the Chronic Care Model
Source: J Gen Intern Med. 2025 Jun 6;41(4):913–21. doi: 10.1007/s11606-025-09641-0 (PMC13009416; doi:10.1007/s11606-025-09641-0)
Supplement: Supplementary file 2 — Supplementary file2 (DOCX 2343 KB) [file 11606_2025_9641_MOESM2_ESM.docx]

**Supplemental Materials**

Contents

[**Supplemental Table 1. Mental Health Condition: International Classification of Diseases Codes Visit Diagnoses and Date of Release of Clinical Decision Support Tool** 1](#_Toc185065548)

[**Supplemental Figure 1. Example of 1-page Clinical Decision Support Tool for Depression, Anxiety, ADHD, and PTSD** 2](#_Toc185065549)

[**Supplemental Figure 2. Changes in Mental Health Visit Diagnoses in Primary Care Before and After Primary Care-Behavioral Health Integration Program by Sex** 7](#_Toc185065550)

[**Supplemental Figure 3. Changes in Mental Health Visit Diagnoses in Primary Care Before and After Primary Care-Behavioral Health Integration Program by Birth Cohort** 7](#_Toc185065551)

[**Supplemental Figure 4. Changes in Mental Health Visit Diagnoses in Primary Care Before and After Primary Care-Behavioral Health Integration Program by Race/Ethnicity** 7](#_Toc185065552)

[**Supplemental Table 2. Trends in Mental Health Treatment Before and After Primary Care-Behavioral Health Integration Program (BHIP)** 8](#_Toc185065553)

# **Supplemental Table 1. Mental Health Condition: International Classification of Diseases Codes Visit Diagnoses and Date of Release of Clinical Decision Support Tool**

| Mental Health Condition | ICD-9 and ICD-10 Codes | Clinical Decision Support Release Date |
| --- | --- | --- |
| Any mental health diagnosis | 291-292, 295-298, 300-316  F10-F69, F90-F99 | n/a |
| Depression | 292.6, 296.3, 300.4, 311  F32, F33, F34.1 F38.1 | September 2015 |
| Anxiety | 300.0, 300.2, 300.3  F40-F42 | February 2018 |
| Panic | 300.01, 300.21, 308.0  F40.01, F41.0, F43.0 | July 2016 |
| Attention Deficit Hyperactivity Disorder | 314  F90 | March 2018 |
| Post-Traumatic Stress Disorder | 309.81  F43.1 | December 2018 |

# **Supplemental Figure 1. Example of 1-page Clinical Decision Support Tool for Depression, Anxiety, ADHD, and PTSD**

1. Depression


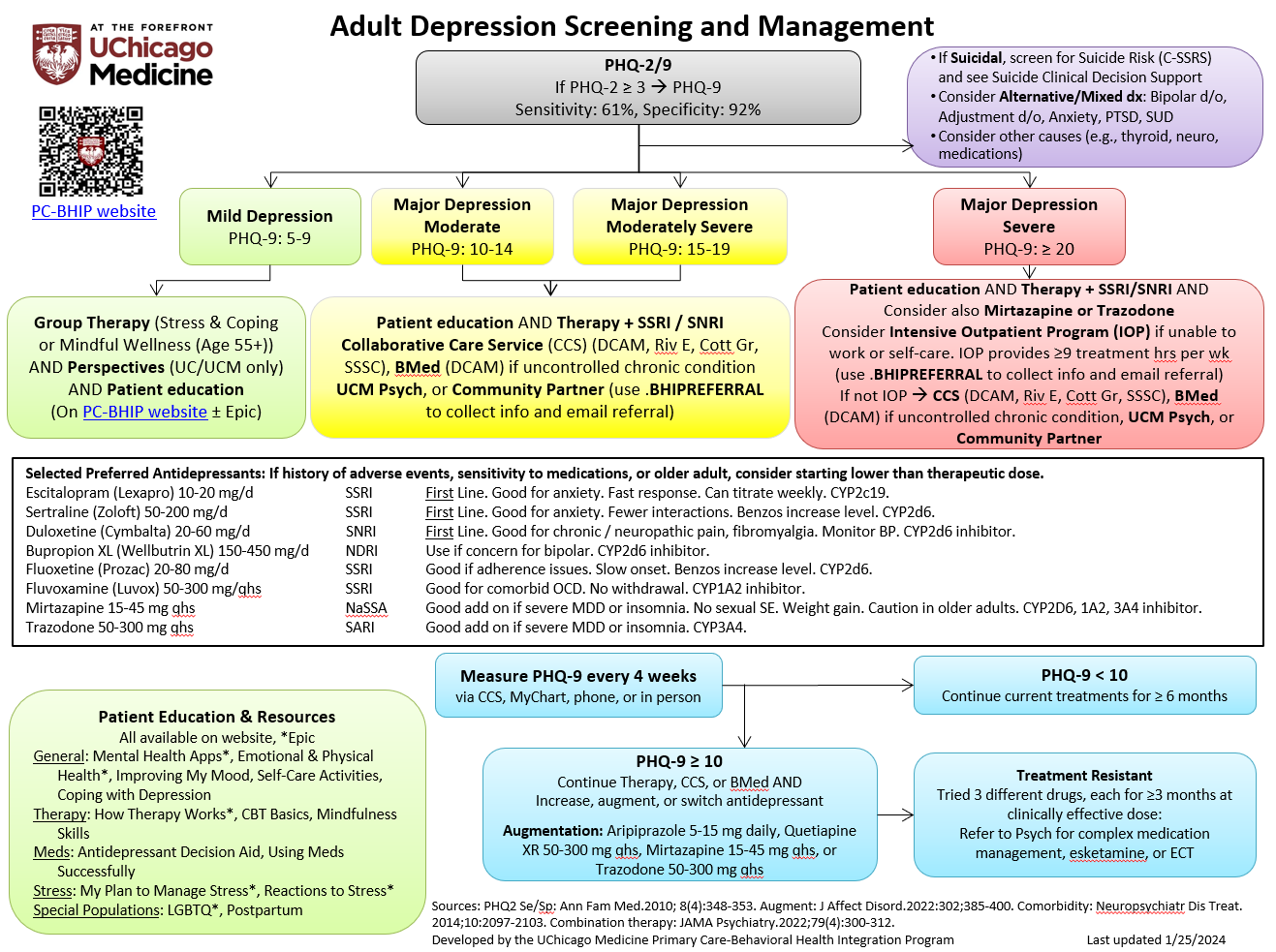


1. Anxiety


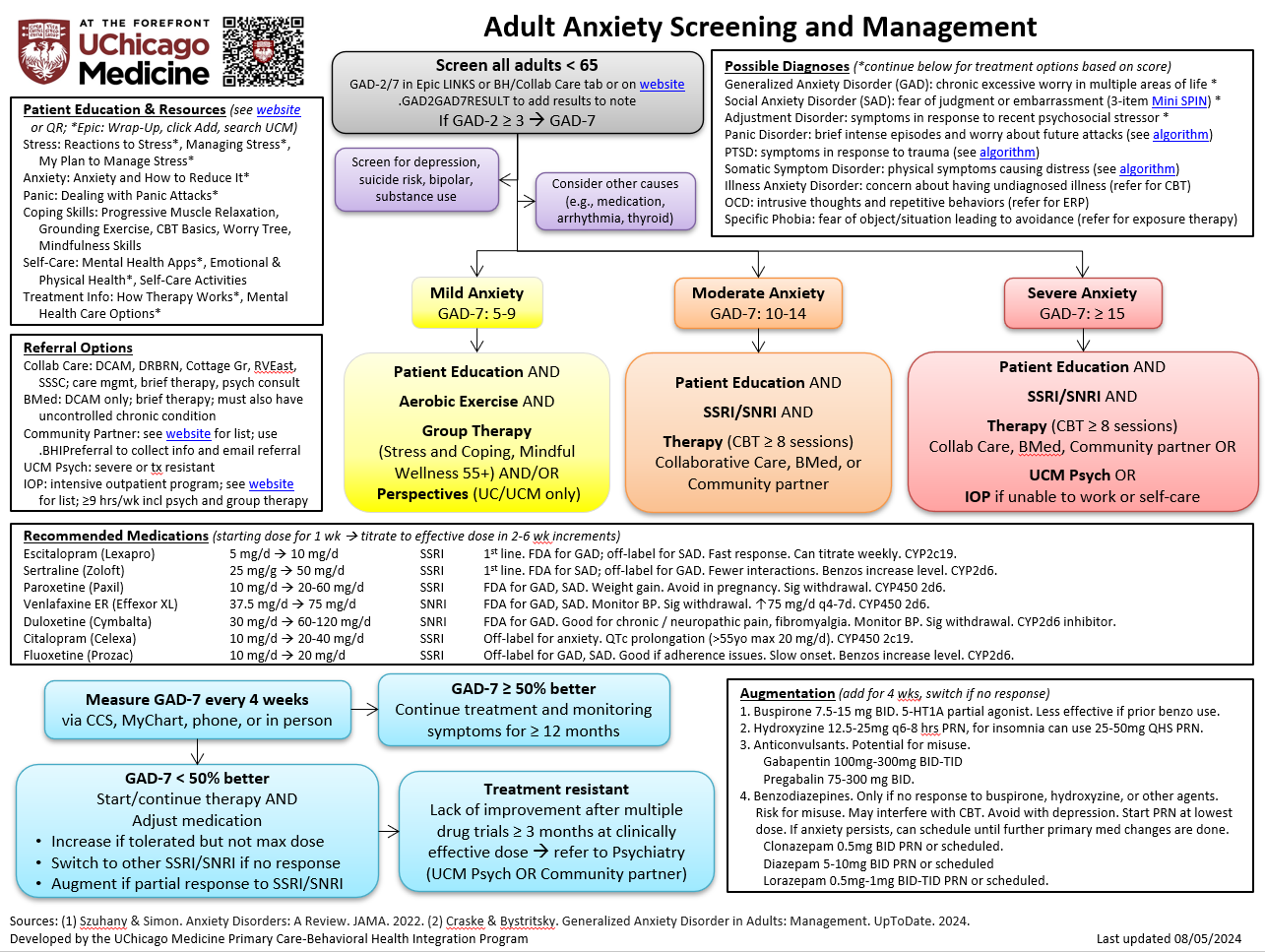


1. Panic


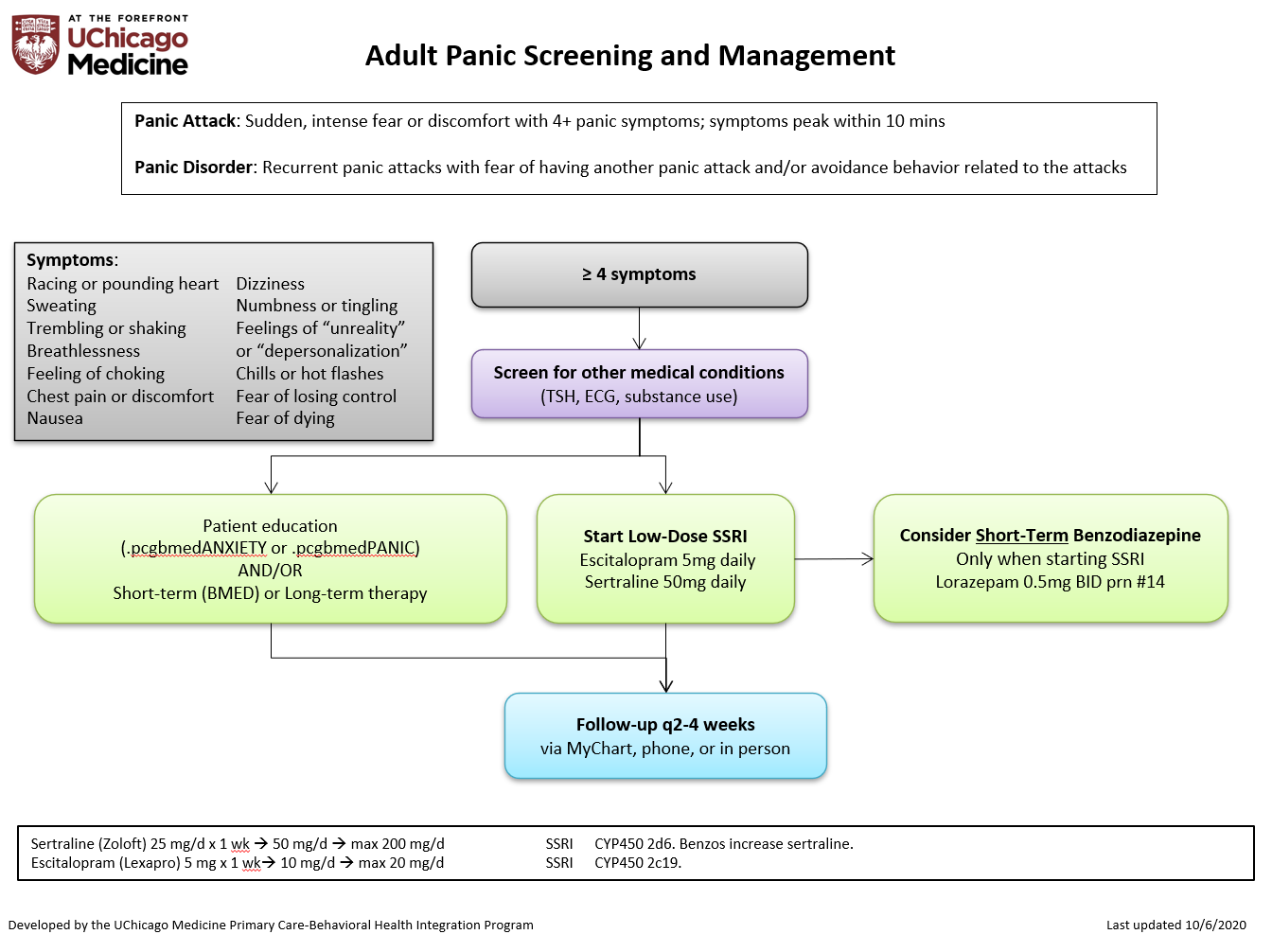


1. ADHD


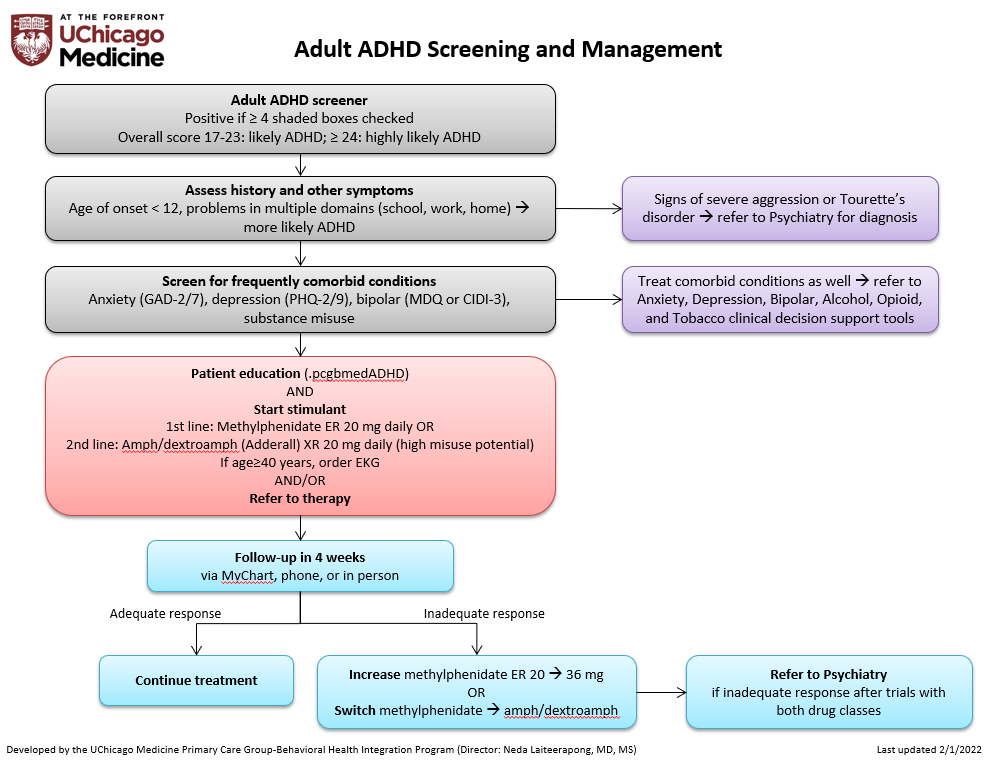


1. PTSD


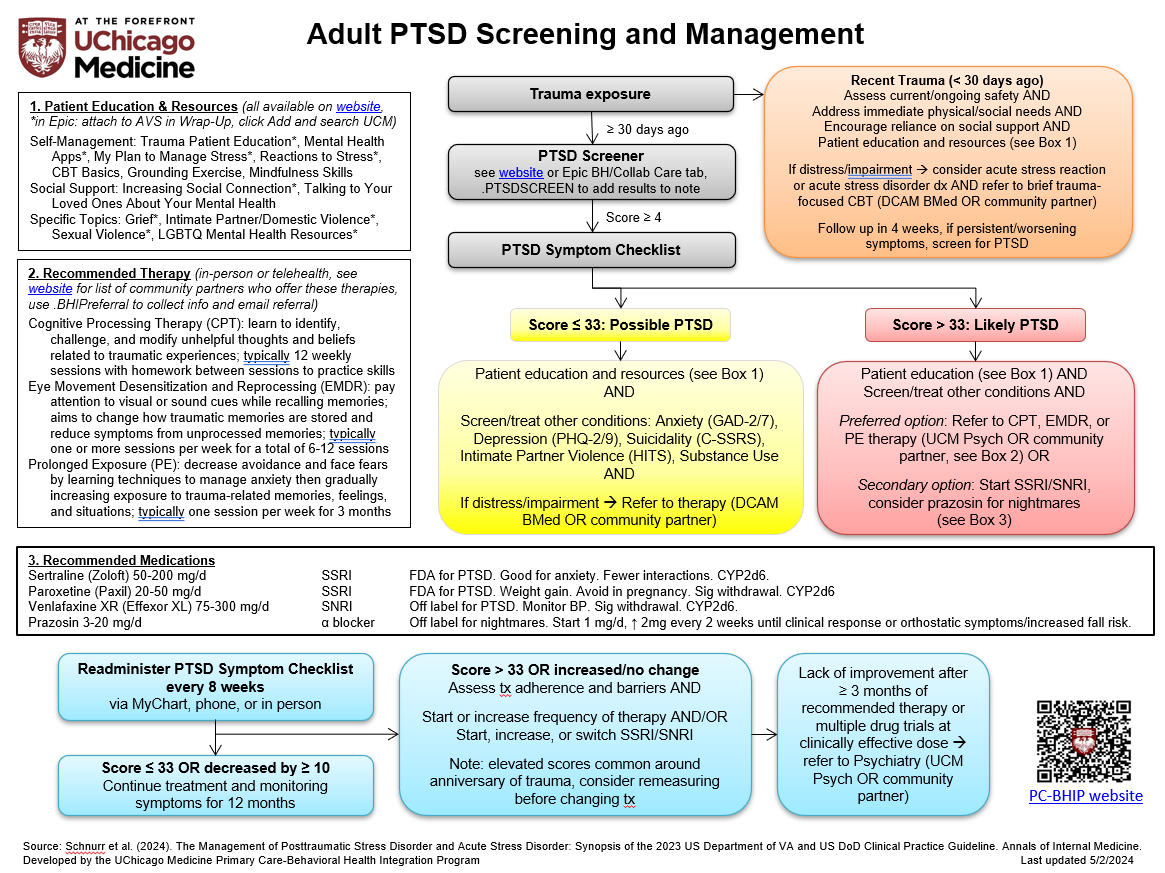


# **Supplemental Figure 2. Changes in Mental Health Visit Diagnoses in Primary Care Before and After Primary Care-Behavioral Health Integration Program by Sex**

Black color: Female; Gray color: Male

Dashed line: Trend before Primary Care-Behavioral Health Integration Program

Solid line: Trend after Primary Care-Behavioral Health Integration Program


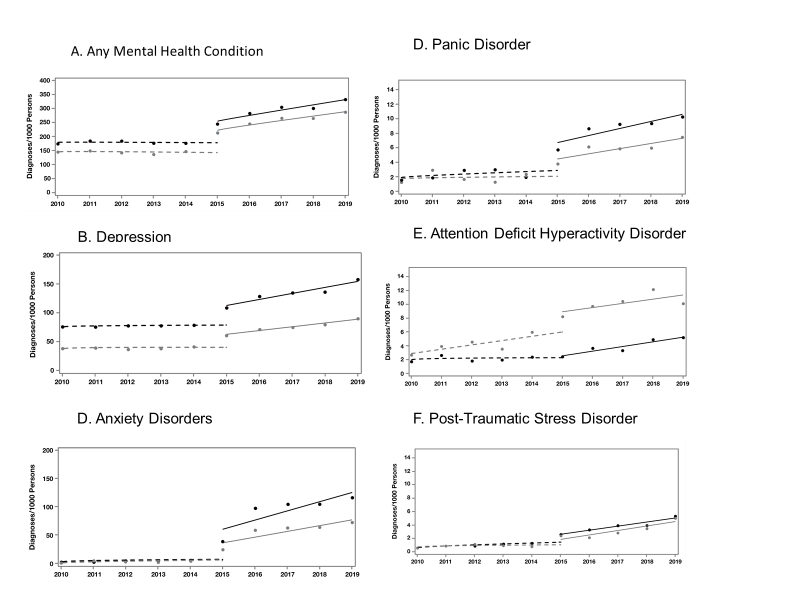


# **Supplemental Figure 3. Changes in Mental Health Visit Diagnoses in Primary Care Before and After Primary Care-Behavioral Health Integration Program by Birth Cohort**

Purple: <1950; Orange: 1951-1974; Green: >=1975

Dashed line: Trend before Primary Care-Behavioral Health Integration Program

Solid line: Trend after Primary Care-Behavioral Health Integration Program


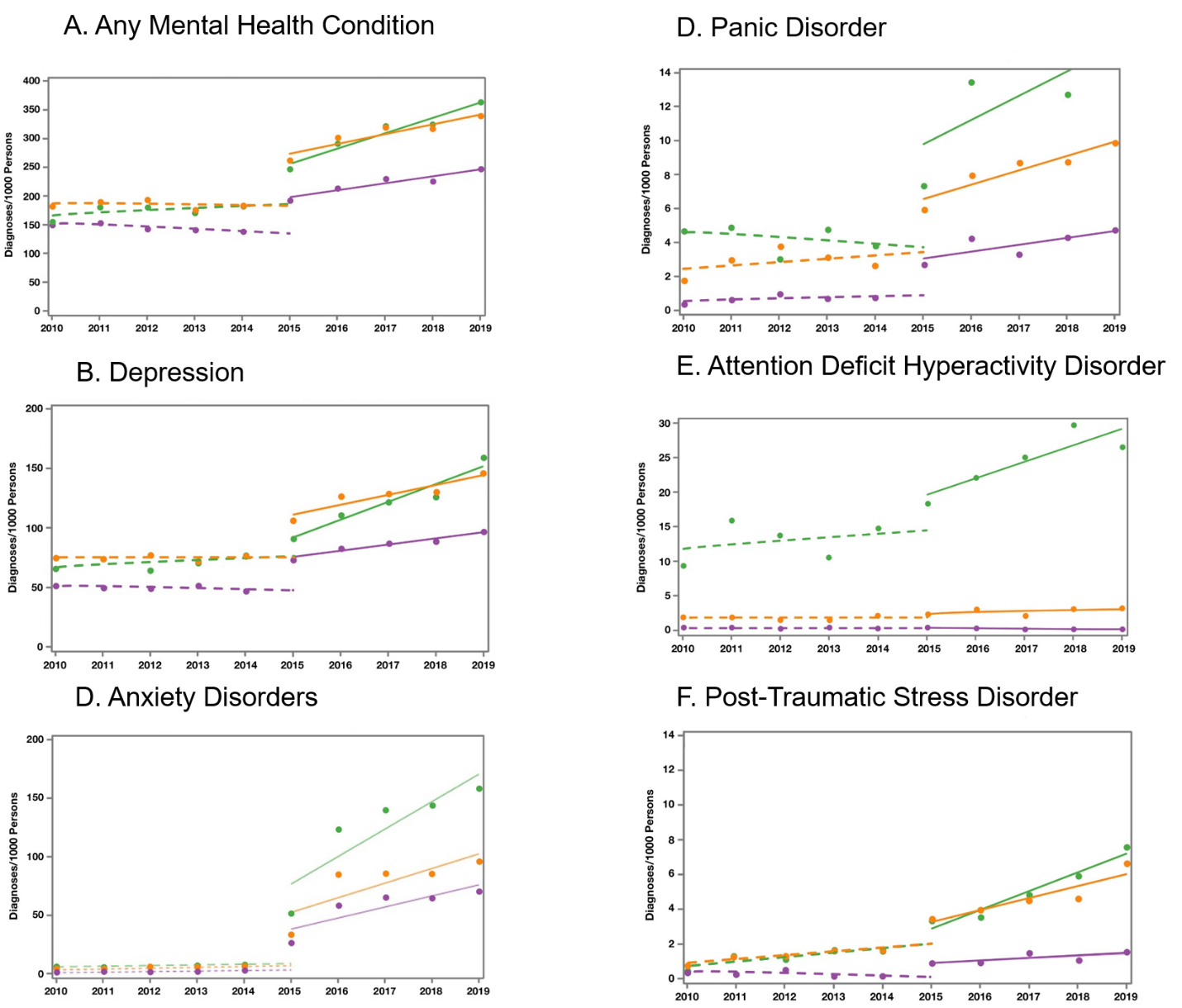


# **Supplemental Figure 4. Changes in Mental Health Visit Diagnoses in Primary Care Before and After Primary Care-Behavioral Health Integration Program by Race/Ethnicity**

Green: African American; Gold: White; Purple: Hispanic; Asian: Red; Blue: Other

Dashed line: Trend before Primary Care-Behavioral Health Integration Program

Solid line: Trend after Primary Care-Behavioral Health Integration Program


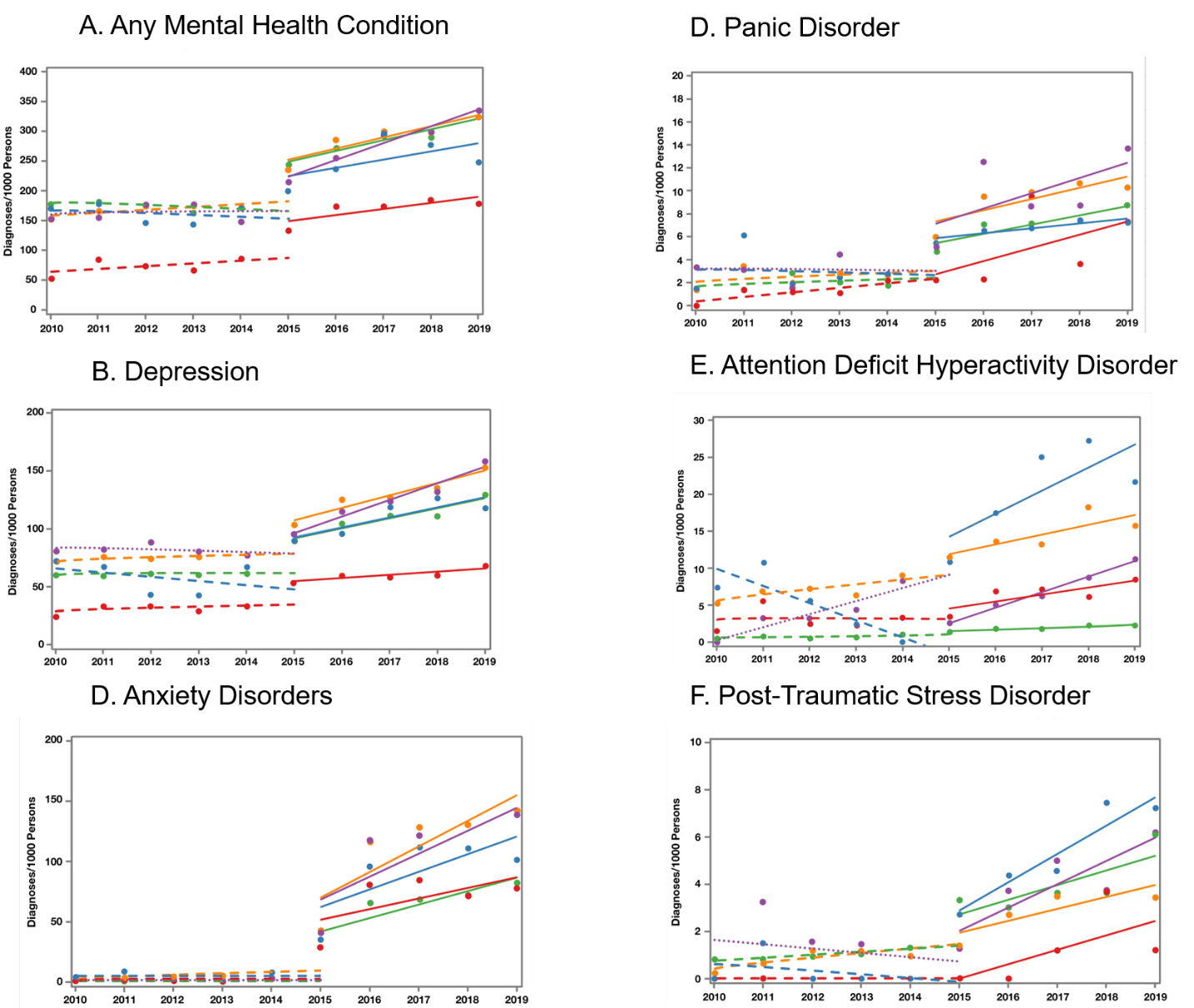


# **Supplemental Table 2. Trends in Mental Health Treatment Before and After Primary Care-Behavioral Health Integration Program (BHIP)**

|  | **Mean Rate (2010-2014)** | **Mean Change Per Year Before PC-BHIP (2010-2014)** | | **Mean Change In Year of PC-BHIP Implementation (2015)** | | **Mean Change Per Year After PC-BHIP (2016-2019)** | |
| --- | --- | --- | --- | --- | --- | --- | --- |
|  | Per 1000 person-years | Per 1000 person-years | P-value | Per 1000 person-years | P-value | Per 1000 person-years | P-value |
| **Follow-up in Primary Care** | 792.3 (732.8-851.8) | -40.6 (-57.6 to - 23.6) | 0.003 | 102.1 (32.0-172.2) | 0.03 | 20.9 (3.9-37.9) | 0.05 |
| **Referral to PCBH** | - | - | - | 117.9 (69.8-166.0) | 0.003 | 10.0 (-1.7-21.7) | 0.14 |
| **Referral to Psychiatry** | 304.4 (249.8-359.1) | 43.1 (27.5-58.7) | 0.002 | -38.7 (-103.1-25.7) | 0.28 | -59.8 (-75.5 to -44.2) | <0.001 |
| **Initiation of Psychiatric Medication** | 79.6 (19.1-140.2) | 52.0 (34.7-69.3) | 0.001 | -48.7 (-120.0-22.6) | 0.23 | -3.8 (-21.1-13.5) | 0.68 |
